# Robotic Surgery in Gynecologic Oncology

**DOI:** 10.1155/2011/139867

**Published:** 2011-11-16

**Authors:** Robert DeBernardo, David Starks, Nichole Barker, Amy Armstrong, Charles A. Kunos

**Affiliations:** ^1^Division of Gynecologic Oncology, Departments of Obstetrics and Gynecology, University Hospitals Case Medical Center and Case Western Reserve School of Medicine, Cleveland, OH 44106, USA; ^2^Department of Radiation Oncology, CASE Comprehensive Cancer Center, University Hospitals Case Medical Center and Case Western Reserve School of Medicine, 11100 Euclid Avenue, LTR 6068, Cleveland, OH 44106, USA

## Abstract

Robotic surgery for the management of gynecologic cancers allows for minimally invasive surgical removal of cancer-bearing organs and tissues using sophisticated surgeon-manipulated, robotic surgical instrumentation. Early on, gynecologic oncologists recognized that minimally invasive surgery was associated with less surgical morbidity and that it shortened postoperative recovery. Now, robotic surgery represents an effective alternative to conventional laparotomy. Since its widespread adoption, minimally invasive surgery has become an option not only for the morbidly obese but for women with gynecologic malignancy where conventional laparotomy has been associated with significant morbidity. As such, this paper considers indications for robotic surgery, reflects on outcomes from initial robotic surgical outcomes data, reviews cost efficacy and implications in surgical training, and discusses new roles for robotic surgery in gynecologic cancer management.

## 1. Introduction

Management of gynecologic cancer often involves surgery followed by radiation, chemotherapy, or a combination of both therapies. It is important for the gynecologic oncologist to consider technical aspects of surgery as it pertains to a patient's goals for surgical intervention, planned extent of surgical removal of cancer-bearing organs and tissues, a patient's postoperative speed of recovery, and how these relate to the timing and administration of future anticancer therapies. Techniques of minimally-invasive surgery, initially involving laparoscopy and more recently robot-assisted surgery, have emerged to address these considerations [1–3]. Early on, gynecologic oncologists found that laparoscopic surgery was associated with less surgical morbidity and shortened postoperative recovery. Robotic surgery has expanded the potential cohort of women capable of undergoing minimally-invasive surgery, now cautiously including the morbidly obese, those in poor health, and those having numerous comorbidities [4–7].

Early clinical successes of robotic surgery in the management of gynecologic cancers have prompted gynecologic oncologists to consider this procedure more often. Here, we discuss use of robotic surgery specifically for gynecologic cancer management, focusing on its applications in the management of cervical, endometrial, and ovarian cancers.

## 2. Technical Aspects of Robotic Surgery in Gynecologic Cancer Management

**Figure 1:**
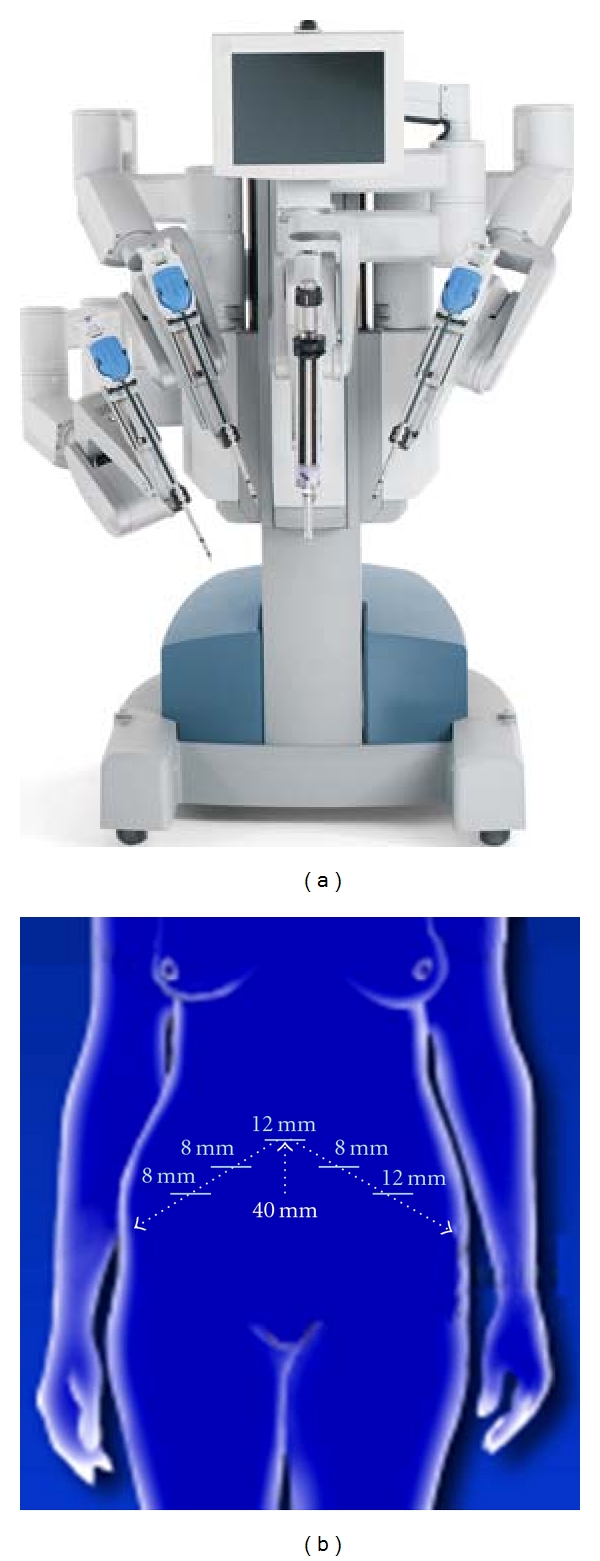
Robotics in gynecologic cancer surgery. (a) Depicted is a da Vinci robotic surgical platform used at University Hospitals of Cleveland (Cleveland, Ohio). (b) With the patient in dorsal lithotomy position and with the robot docked between the legs, an initial 12-millimeter (mm) port incision is made 40 mm cephalad to the umbilicus. Additional 8-millimeter port incisions are made following a conventional triangle arrangement. A 12-millimeter instrument port for an assistant is also made as indicated. Stereoscopic optics in the surgeon control console allows for three-dimensional viewing of the surgical field (not shown).

Robotic surgery differs substantially from laparoscopic surgery in important ways. Conventional laparoscopy utilizes a two-dimensional camera with images projected to monitors positioned in proximity to the surgeon within the operating room. Surgery is performed through 5-to-12-millimeter incisions through which a camera and rigid instruments are placed through abdominal ports and controlled directly by the surgeon at the surgical bedside. Commonly listed limitations to conventional laparoscopy are difficulty in manipulating the instruments and collapsed two-dimensional optics rendering complex tasks associated with more radical pelvic surgery arduous. While an experienced laparoscopic surgeon may be able to accomplish radical hysterectomy with or without lymphadenectomy using laparoscopic instrumentation, the nontraditional skills and unfamiliarity with two-dimensional optics needed for laparoscopy have led to infrequent use of a laparoscopic approach by gynecologic oncologists [8]. As such, the number of patients benefitting from a minimally-invasive procedure to manage their gynecologic cancer is low. With the introduction of robotics, many of the frustrations and limitations of inexperienced laparoscopic surgeons have been minimized due to the improvement in ergonomics inherent in the robotic platform [4–23]. Using a robotic platform to perform surgery allows the primary surgeon to control surgical instruments (i.e., up to three surgical instrument arms plus camera) in a “hands-off” manner (Figure 1). Moreover, the surgical instruments have greater range of motion than conventional laparoscopic instrumentation, allowing “wristed action” rotation of instruments and motion scaling. Improved optics allow three-dimensional view of the surgical field. These technical aspects of robotic surgery have advantages of speeding learning new surgical skills by the surgeon and translating and adapting their own surgical skills to the robotic surgery platform. Descriptions of various robotic-assisted surgical techniques are referenced for the reader [4–23].

Robotic surgery platforms have evolved from the initial telesurgery units perfected for the military [3] to voice-operated laparoscopy such as the automated endoscopic system for optimal positioning (AESOP, Computer Motion Inc. [Goleta, Calif]) [4, 9] to robotic platforms. The two most common robotic platforms are the ZEUS (Computer Motion Inc. [Goleta, Calif]) [10] and the da Vinci (Intuitive Surgical, Inc. [Sunnyvale, Calif]) [11] systems. Both of the robotic platforms position the gynecologic oncologist at a console remote from the patient undergoing surgery. Although the surgeon is no longer at the bedside, the three-dimensional optics, dexterity provided by wrist-like instrument rotation, reduction in surgeon hand tremor, and motion scaling have peaked interest among gynecologic oncologists to do cancer surgeries on a minimally-invasive robotic device. As an example, Figure 1 provides an example of the da Vinci robotic surgery platform in use at University Hospitals of Cleveland (Cleveland, Ohio).

## 3. Robotic Surgery for Cervical Cancer

For many gynecologic oncologists, it remains an open question whether robotic-assisted minimally-invasive surgery can be substituted for conventional laparotomy in all gynecologic cancer patients. Outside of clinical trials, consideration for goals of surgical intervention, patient recovery, and adequate assessment of risk factors for local or distant recurrence is needed. From available data in cervical cancer patients (Table 1), robotic surgery appears to provide sufficient surgery to assess pathologic tumor size, tumor grade, deep cervix organ invasion, lymphovascular invasion, cancerous lymph node status, and cancer-free margins of resection without undue risk of intraoperative injury. For this discussion here, a comprehensive surgicopathological staging procedure comprised removal of the uterus and ovaries, adnexa, and any number of lymph nodes. Such a procedure was achieved in the majority of patients undergoing robot-assisted surgeries (Table 1). As the basis of adjuvant radiation and chemotherapy recommendations are founded in surgicopathological parameters, gynecologic oncologists who perform robot-assisted radical hysterectomy for cervical cancer must ensure that their robotic surgery continues to provide this informative data. 

Experiences with robotic surgery to perform radical hysterectomy in patients with early stage cervical cancer have demonstrated feasibility and safety of the technique (Table 1). Blood loss secondary to cutting and extirpation of the uterus and cervix appears minimal when performing radical hysterectomy for cervical cancer. Patient in-room operative times are longer than a conventional approach; however operative times do decrease with increasing familiarity and robotic skill. To date, robotic and laparoscopic procedures are associated with fewer lymphocysts, lymphoceles, postoperative infections, and ileus [12–15]. This has contributed to a widespread adoption of robotic surgery in the management of women with early cervical cancer. Since improved cervical cancer screening has led to the earlier detection of organ-confined disease, it is likely that minimally-invasive robotic surgery will become more commonplace in the management of early-stage cervical cancer. 

Clinical use of robotic surgery for management of more bulky (>4 cm) cervical cancer remains sparse (Table 1). Moreover, there are no randomized studies evaluating robotic surgeries compared to laparoscopy-assisted surgeries or conventional laparotomy. Initial case experience for early-stage cervical cancer is encouraging. However, caution is warranted as there have not yet been sufficient studies of port and operative site relapse rates (Table 1). Further study is ongoing to assess these important questions.

## 4. Robotic Surgery for Endometrial Cancer

Gynecologic oncologists were quick to recognize the advantages of robotic-assisted surgery in women with endometrial cancer. Initial studies praised ease of surgical technique, adequacy of surgical specimens for cancer staging, and reduction in patient hospital stay and time to recovery [8, 16–20]. One particular advantage of the robotic platform was surgical confidence in adequate lymphadenectomy (i.e., >4 lymph nodes retrieved from right and left pelvis and para-aortic node-bearing tissues) without undue risk of injury to pelvic organs and blood vessels ([8, 16–20], Table 2). 

Consensus definitions of adequacy of cancer-staging surgery remain debatable. Endometrial cancer data have indicated that high tumor grade, deep myometrial invasion, involvement of the cervix, lymphovascular invasion, and presence of malignant lymph nodes, all contribute to adjuvant treatment recommendations [24]. There has been no indication that robotic surgery limits these assessments (Table 2). An important retrospective study comparing robotic-assisted surgery and conventional surgery backs this claim [8]. Of 275 women undergoing minimally-invasive total hysterectomy and pelvic and para-aortic lymphadenectomy, 102 underwent robotic-assisted and 173 underwent conventional laparoscopic cancer-staging surgeries. Surgery performed did not bias cancer grading or tumor type, number of lymph nodes retrieved from the pelvis or para-aortic tissues, or excised uterine weight. Intraoperative injury rates were similar (2.0% robotic versus 3.5% laparoscopic, *P* = 0.71). There were also no substantial trends in prolonged hospital stay (1.9 days versus 2.3 days, *P* = 0.09) or requirement of second surgery (e.g., small bowel perforation or repair of vaginal apex dehiscence, 1.9% versus 1.2%) after robotic or conventional surgery, respectively. Overall, robotic-assisted surgeries were deemed safe and comparable to laparoscopic surgeries. While these results are impressive, this study consisted of cases done by a single surgeon at a single academic practice dedicated to improving minimally-invasive surgical techniques, introducing substantial selection and performance bias. Over multiple studies, there has been a trend for more vaginal apex dehiscence in women undergoing robotic-assisted surgical procedures, with rates of 2.9% after robotic and 2.4% after conventional surgery [16–20]. In addition, rates of procedure conversion from robot assisted to laparotomy have ranged between 4% and 15% [16–20]. Dedicated multi-institutional study of robotic-assisted surgical approaches for endometrial cancer is needed so that over- or underestimates of appropriateness of cancer staging, surgical complications, and operative and patient recovery time are more relevant to practicing gynecologic oncologists. 

Moreover, it is important for the gynecologic oncologist to recognize that surgery in the morbidly obese presents a unique surgical challenge. Not only are these patients more susceptible to postoperative complications such as poor wound healing, but their body mass often makes the surgical procedure technically more challenging whether the approach is laparoscopic or open. Many feel that the advantages of a robotic platform help overcome some of the barriers to operating on the morbidly obese with endometrial cancer. To date, few papers have addressed the use of robotic surgery specifically in the obese population. The limited data to date has shown that increased body mass index is not generally associated with greater complications in robotic staging for endometrial cancer [25]. A randomized study conducted by the Gynecologic Oncology Group (LAP-2) comparing laparoscopy and laparotomy showed no substantial differences in oncologic assessment or outcome with laparoscopy, but there were increased odds of not successfully completing laparoscopy without conversion to laparotomy in the obese (odds ratio: 1.11, 95% confidence interval 1.09 to 1.13) [26]. While it remains controversial to use robotic-assisted procedures in the obese, it has been shown that surgical intervention followed by adjuvant therapy successfully manages pelvis-confined endometrial cancer in the morbidly obese [27, 28]. Indeed, these studies indicate that the morbidly obese patient does not often have cancer limit life expectancy, but rather comorbidities resulting from obesity contribute to mortality. Robotic surgical techniques that limit confounding surgical morbidity in the obese may be of interest to the gynecologic oncologist. Further surgical development of robotic-assisted instrumentation for the obese is expected.

## 5. Robotic Surgery for Ovarian Cancer

Management of epithelial ovarian cancer is predicated upon optimal cytoreductive surgery, with less than 1 cm of residual disease. Recurrence and overall survival improves with further cytoreduction incrementally, with microscopic residual disease followed a platinum-taxane combination chemotherapy infusion [29] results in the greatest overall and progression-free survival. Most commonly ovarian cancer is identified at an advanced stage often requiring radical surgical procedures to achieve “optimal” status. Minimally-invasive surgeries for maximal cytoreduction of ovarian cancers have been attempted since the 1990s. Whether robotic-assisted surgery improves upon the ability to surgically cytoreduce ovarian cancer is an open question. While the improved ergonomics may aid in these type radical surgery, other limitations inherent in the robotic platform—namely the inability to simultaneously operate in the pelvis and abdomen—remain a significant disadvantage. 

Robotic surgery for ovarian cancer management remains relatively untested (Table 3). In the limited experience to date, blood loss and postoperative complications of bowel injury and wound dehiscence are infrequent (Table 3). Port site relapses have not been reported routinely among the early investigational studies. A single manuscript has compared robotic surgery to traditional means of ovarian cancer staging surgery. In this case control study, 25 patients undergoing primary staging for epithelial ovarian cancers were compared to similar patients treated by laparoscopy and laparotomy. The authors concluded that laparoscopic or robotic surgery is reasonable approach for primary tumor excision in patients with ovarian cancers. It must be emphasized that these patients were highly selected and their results are not likely to apply to all patients with ovarian cancer. The authors themselves noted that for patients with advanced disease requiring multiple complicated additional procedures, laparotomy remains the optimal surgical approach [23].

Clinical trials have demonstrated that intraperitoneal routes of chemotherapy administration should be strongly considered for treatment of women with intrabdominal spread of ovarian cancer. While one advantage of robotic surgery is that postoperative surgery-related complications and patient recovery time may be reduced, reducing wait time for chemotherapy, the technique may present a critical shortcoming in that the port sites for the robotic surgical instruments may be seeded with tumor. While this has yet to be rigorously investigated, concerns that effectiveness of intraperitoneal and intravenous chemotherapies may be lowered due to this phenomena. To this end, the use of robotic surgery in the setting of ovarian cancer management is not recommended until further study suggests otherwise.

## 6. Robotic Surgery in Anticipation of Radiation Therapy

Most often, surgery for gynecologic malignancies involves removal of pelvic organs in the female reproductive system precluding subsequent pregnancy. However, there are clinical situations such as adolescent female lymphoma or cervical cancer in which fertility-sparing surgery and tumor-directed radiation may be considered [25, 29–35]. To shield the ovaries from irradiation, an oophoropexy or ovarian transposition may be performed either laparoscopically or robotically. Here, minimally-invasive robotic surgery may play a role as demonstrated in cases of cervical cancer management. Transposition of the ovaries to midline or to lateral iliac wings, depending on the radiotherapeutic target, results in radiation dose to the ovaries of 4% to 8% of the pelvic radiation dose [36–39]. When done, ovarian position should be marked with surgical clips that can be identified on radiation therapy imaging. While ideal surgical removal of gynecologic cancers will often limit such a role for robotic surgery, the ability of robotic surgery to preserve fertility should not be overlooked when radiation therapy may be contemplated.

## 7. Training of Surgeons in Robotic Surgery

Hysterectomy and gynecologic organ surgery are among the most common services surgeons provide for American women besides cesarean delivery [40]. When surveyed, surgeons have increased their use of robotic-assisted procedures from 10% in January 2008, to 40% in February 2011 according to one source [41]. Such a trend implies that surgical training in robotic surgery become in line with other fundamental aspects of surgical residency, and perhaps because of its perceived importance by patients, a skill demanded for maintenance of surgical certification. To this end, an effective platform for teaching this skill may hinge upon didactic symposia and clinical dry laboratory practice. 

A robotic platform improves positional ergonomics and visualization for training physicians except perhaps among the most experienced laparoscopic surgeons. Surgical operating room time and effort by the surgeon decreases over time and with gained experience. It has been suggested that 12 cases are needed by a surgeon and ancillary staff to develop the orchestrated effort for facile robotic surgery—operating room times dropped from a mean of 410 minutes for the first 12 robot-assisted cases to a mean of 337 minutes for the next consecutive 12 robot-assisted cases in one series [41]. Such data argue for a dedicated hospital-based surgical robotics team to reduce operative time, global room time, and (non)renewable resources. Moreover, training of surgical residents and inexperienced surgeons in the techniques of robotics may protract both operative and global room time in some instances, but the overreaching goal of mastering a surgical skill demanded in their future practice mandates patience at the console by the learned surgeon during such instruction. 

More measures for mastery of robotic skill include (a) complexity of surgical case undertaken with robotics and (b) conversion rates from robotics to laparotomy. A surgical training beginning point for pelvic robotic surgery may be the performance of a hysterectomy, which must be learned from abdominal, vaginal, and laparoscopic approaches. Adding a robotic-assisted approach to the learning surgeon's repertoire seems a logical first step. In one program, surgeons new to robotic surgery had a steep learning curve for robotic-assisted suturing of the vagina, where mean robot console times of 326 seconds versus a mean laparoscopic suturing time of 382 seconds was observed among 12 residents in training [41]. Data are still emerging regarding the complexity of surgical care provided by robotic platforms. Conversion rates from robotics to laparotomy range from 3% to 12%, doing so for a multitude of unspecified reasons in most series [15, 41, 42]. 

Lawful cause of action claims against a surgeon for insufficient training or credentialing and lack of patient informed robotic surgical consent have been filed [43]. For many surgeons currently in practice, robotic surgery developed after completing their residency training. The practitioner must appreciate that to counsel a woman on robotic surgery, one must be intimately familiar with the inherent hazards of a planned robotic surgery. Indeed, prior patient knowledge both on a surgeon's familiarity and performance of robotic surgery and the intrinsic complications that may arise from a robotic surgery approach may affect a woman's decision to undergo a robotic-assisted surgery. This is not to imply that procedural hazards do not arise more or less with any particular surgical approach, but rather it is to bring attention to physician duty to inform a woman about anticipated risks and mitigation of risks during a robotic-assisted surgery. It is critical that the legal pitfalls arising from insufficient training, credentialing, and informed consent should be addressed [43].

## 8. Robotic Surgery Costs for Oncologic Procedures

Robot-assisted gynecologic surgery costs more than conventional laparoscopic procedures [44]. Publications on the cost effectiveness of oncologic robotic surgery do not yet include persuasive and informative financial data supporting or refuting robotic-assisted procedure. In a single American institution cost review of consumables, operating room time, and anesthesia time [41], it was found that a robotic hysterectomy cost of $18,570 (US$) was billed compared to a laparoscopic hysterectomy cost of $13,867 (US$). Capital investment in a robotic surgery platform was not accounted for in this analysis. Consider that the price tag for a robotic platform ranges from $1.2 to $1.5 million US dollars and comes with a yearly maintenance fee of $138,000 (US$). Indeed, the use of robotics may be more expensive in current dollars than performing the same procedure either by laparoscopy or even by laparotomy. And yet, “cost savings” are created by an offset of reduced hospitalization and resources, lower costs associated with management of resultant surgical morbidity, and earlier patient return to the workforce. Data remain immature for full comment. Financial impacts of robotic surgery for oncologic procedures are active arenas for health and marketing research. 

Implementing a robotics program for oncology services in tertiary cancer centers may not give the chance to initiate a program with undemanding cases. A shift from conventional laparotomy and laparoscopy to robotic-assisted procedures may be time consuming and ultimately limited by a surgeon's ability and flexibility to reserve additional operating room time. Anticipated revenue streams may need to be considered adaptive until surgeon and staff efficiency peaks. Quality of life outcomes on the economic impact of robotic-assisted surgery are awaited.

## 9. Expert Commentary and Conclusion

Despite the encouraging early results suggesting minimally-invasive robotic surgery for women with gynecologic cancers, questions remain about the surgical effectiveness of this approach. In small clinical studies, robotic surgery has shown promising results of reduced morbidity. Further study of robotic surgery technical parameters is needed prior to widespread clinical application of robotic surgery in the management of gynecologic cancers. Training programs are now in place. While it is important to investigate alternative means of surgery with high precision, it remains unclear whether robotic surgery can offer the same therapeutic efficacy as laparotomy. Moreover, cost analyses of robotic-assisted surgery versus other surgery are underway. Both enthusiasm and restraint are appropriate in interpreting available robotic surgery data for treatment of gynecologic cancers. In the end, randomized data will be needed to better assess the oncologic outcome of robotic surgery for gynecologic malignancy.

## Figures and Tables

**Table 1:** Robotic surgery in cervical cancer.

Reference	Platform	Patients	Stage	Surgery	Operative time mean (min)	Blood loss mean (mL)	Lymph node count (Mean)	Comprehensive surgicopathologic staging *N* (%)	Complications	Port site relapse *N* (%)
[12]	da Vinci	10	IA1-IB1	Radical hysterectomy	207	355	28	10 (100%)	No conversion to laparotomy	0 of 10 (0%)
[13]	Zeus/da Vinci	18	IA2-IB2	Radical hysterectomy	226	175	26	10 (56%)	1 pneumothorax, 1 pleural effusion	Not reported
[14]	da Vinci	35	IA1-IB1	Radical hysterectomy	264	82	20	35 (100%)	3 cystotomy, 2 lymphocyst, 1 lymphedema	1 of 35 (3%)
[15]	da Vinci	51	IA2-IIA	Radical hysterectomy	211	97	34	46 (91%)	5 (10%) self-catheterization of bladder	Not reported

**Table 2:** Robotic surgery in endometrial cancer.

Reference	Platform	Patients	Stage	Surgery	Operative time mean (min)	Blood loss mean (mL)	Mean lymph node count	Comprehensive surgicopathologic staging *N* (%)	Complications	Port site relapse *N* (%)
[8]	da Vinci	102	Ia–IVa	Radical hysterectomy	237	109	22	102 (100%)	2 bowel injuries, 1 lymphocele, 1 vaginal dehiscence	Not reported
[16]	da Vinci	103	Ia–IIIc	Radical hysterectomy	283	75	33	102 (99%)	1 bowel injury, 2 lymphatic injuries, 0 vaginal dehiscence	Not reported
[17]	da Vinci	56	Ia–IIIc	Radical hysterectomy	177	105	19	56 (100%)	1 ileus, 1 respiratory failure, 4 vaginal dehiscences	Not reported
[18]	da Vinci	85	Ia–II	Radical hysterectomy	242	99	29	85 (100%)	11 (13%) complications, 2 vaginal dehiscences	Not reported
[19]	da Vinci	100	Ia–IIIc	Radical hysterectomy	171	103	19	100 (100%)	5 (5%) complications, 5 vaginal dehiscences	Not reported
[20]	da Vinci	25	Ia–IIIc	Radical hysterectomy	180	67	18	25 (100%)	2 vessel injuries, 2 thrombosis, 2 vaginal dehiscences	Not reported

**Table 3:** Robotic surgery in ovarian cancer.

Reference	Platform	Patients	Stage	Surgery	Operative time mean (min)	Blood loss mean (mL)	Debulk to <2 cm *N* (%)	Comprehensive surgicopathologic staging *N* (%)	Complications	Port site relapse *N* (%)
[22]	da Vinci	1	IV	Liver/diaph ragm excision	137	100	1 (100%)	Not applicable	1 four-day postoperative pleural effusion	0 of 1 (0%)
[23]	da Vinci	25	I–IV	Debulking hysterecomy	315	164	21 (84%)	25 (100%)	2 cystomies, 1 aortic bleed, 2 vaginal dehiscence, 1 ileus	0 of 25 (0%)
